# Fragile lip in a patient with macroglossia due to hemodialysis-associated amyloidosis

**DOI:** 10.1186/s40981-024-00686-4

**Published:** 2024-01-10

**Authors:** Tohru Shiratori, Masahiro Nishimura, Yusuke Horitani

**Affiliations:** 1 Department of Anesthesiology, Ina Central Hospital, 1313-1 Koshiroukubo, Ina, Nagano 396-8555 Japan; 2 Department of Dentistry and Oral Surgery, Nagano Municipal Hospital, 1333-1 Tomitake, Nagano, Nagano 381-8551 Japan; 3 Department of Anesthesiology and Resuscitology, Shinshu University School of Medicine, 3-1-1, Asahi, Matsumoto, Nagano 390-8621 Japan

To the Editor

Lingual amyloidosis is an oral disease occurring in long-term hemodialysis patients [1, 2], who may rarely suffer from macroglossia [3, 4]. While securing airway management is crucial in patients with macroglossia to prevent airway obstruction, lip fragility is slightly noticeable. We herein report a case of oral amyloidosis wherein a lip laceration developed in the middle of oral surgery.

A 56-year-old woman (height, 153 cm; weight, 46 kg) underwent partial resection for macroglossia. She has been on hemodialysis for 28 years due to chronic glomerulonephritis. The patient had multiple joint pain in the shoulders and knees with joint swelling in her fingers. Furthermore, she had histories of surgical repair with local anesthesia for the right trigger finger in the thumb and left carpal tunnel syndrome at 47 and 53 years of age, respectively. At 56 years of age, she had difficulty in eating due to pain caused by tongue swelling. Intraoral examination showed the development of hemispherical tumor-like soft nodules with fragile surfaces in the lateral edges of the tongue (Fig. 1). The patient was scheduled for resection of the nodules in the macroglossia. Preoperative blood examination showed a remarkably increased serum beta-2-microglobulin level of 22.8 (normal range, 0.9–2.0) mg/L. T2-weighted magnetic resonance imaging revealed macroglossia in the tongue (Fig. 2). While Mallampati classification was class IV, the patient had flexible neck motion, normal mandibular size, wide mouth opening, and healthy teeth.

**Fig. 1:**
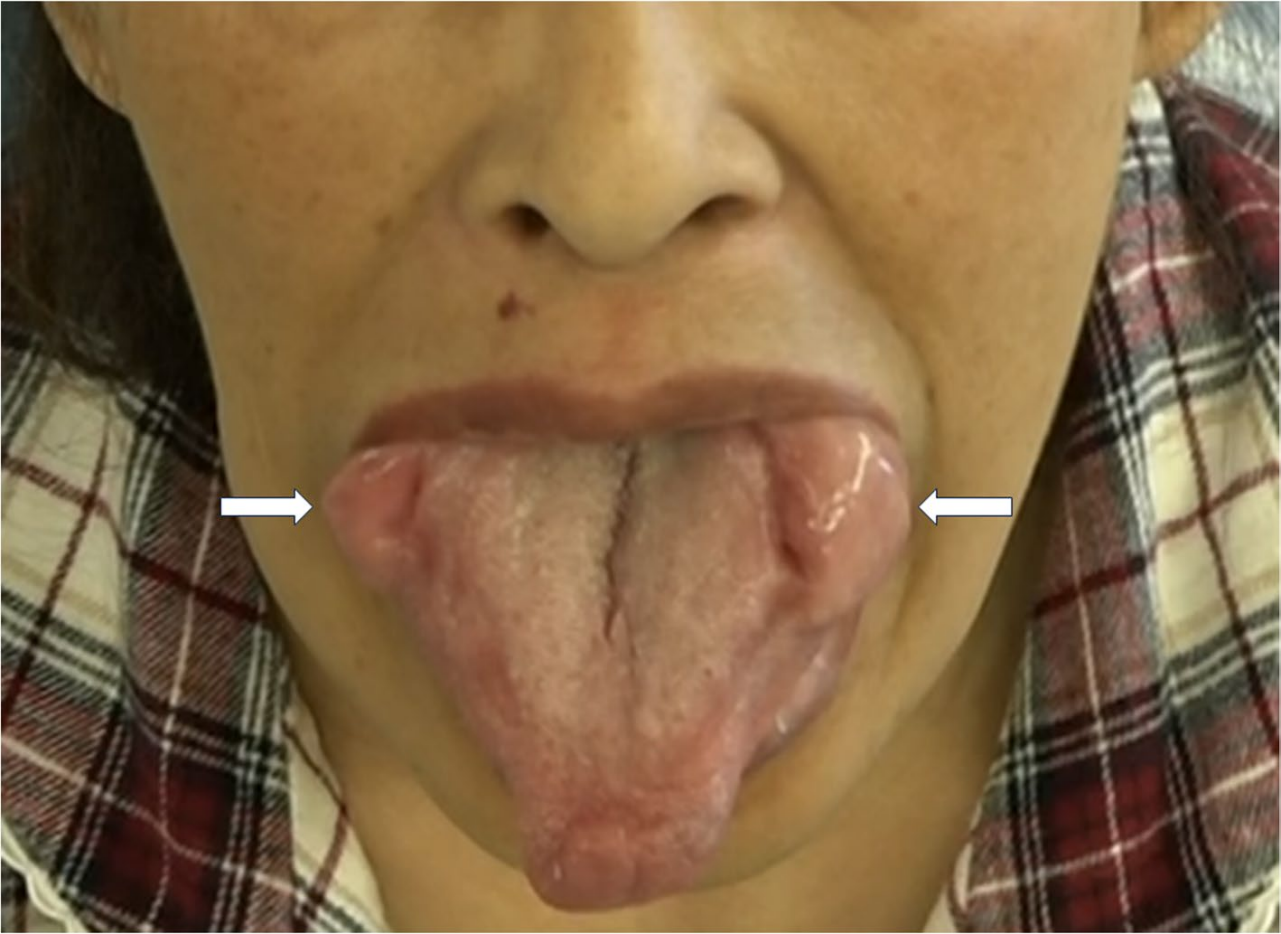
Tumor-like nodules in the lateral edges of the tongue. Intraoral examination revealed macroglossia. White arrows indicate nodules in the bilateral edges of the macroglossia

**Fig. 2:**
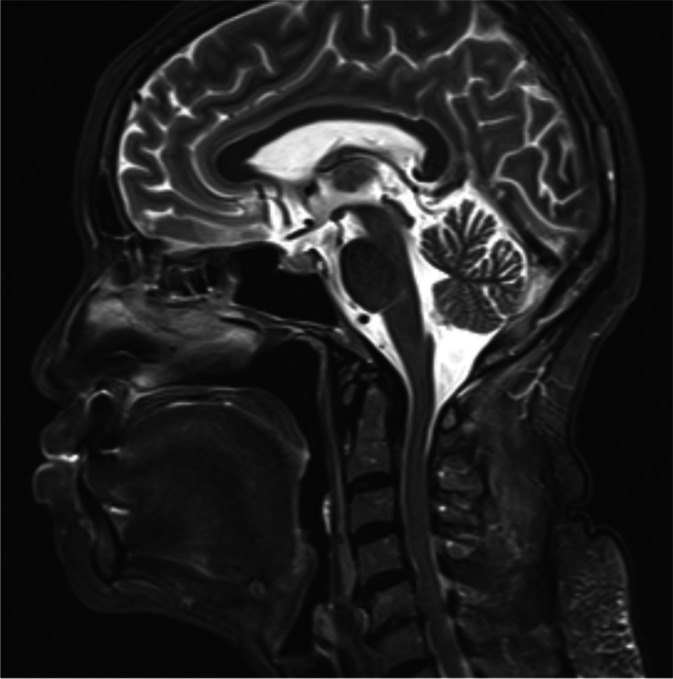
Sagittal view of the head showing macroglossia. T2-weighted magnetic resonance imaging revealed the enlarged tongue. Swelling at the base of the tongue was mild with no airway obstruction

We prepared the equipment for difficult airway management; however, airway management with mask ventilation following anesthetics seemed applicable. After confirming uneventful mask ventilation following propofol administration, rocuronium was administered. Laryngoscopy by an experienced physician showed a Cormack grade of IV. Tracheal intubation was performed using a McGRATH™ MAC videolaryngoscope. The tracheal tube was attached to the right side of the patient’s mouth using two adhesive tapes applied on the ipsilateral cheek. The patient’s lips and tongue were fragile. Compression by steel retractor and mouth gag easily induced hematoma and superficial damage in the tongue and lips. The nodule in the left side of the tongue was resected using an electrical scalpel without hemorrhage. When adhesive tapes were removed to replace the tracheal tube, the upper lip was slightly pulled and a superficial laceration occurred. The transfer of the tracheal tube from the right to the left side of the mouth led to a widening of the wound (Fig. 3). The injured site in the lip was sutured to stop bleeding. Subsequently, the nodule in the right side of the tongue was resected without complications. Postoperative histological examinations of the nodules revealed Congo Red staining-positive amyloid deposits classified as AL(κ).

**Fig. 3:**
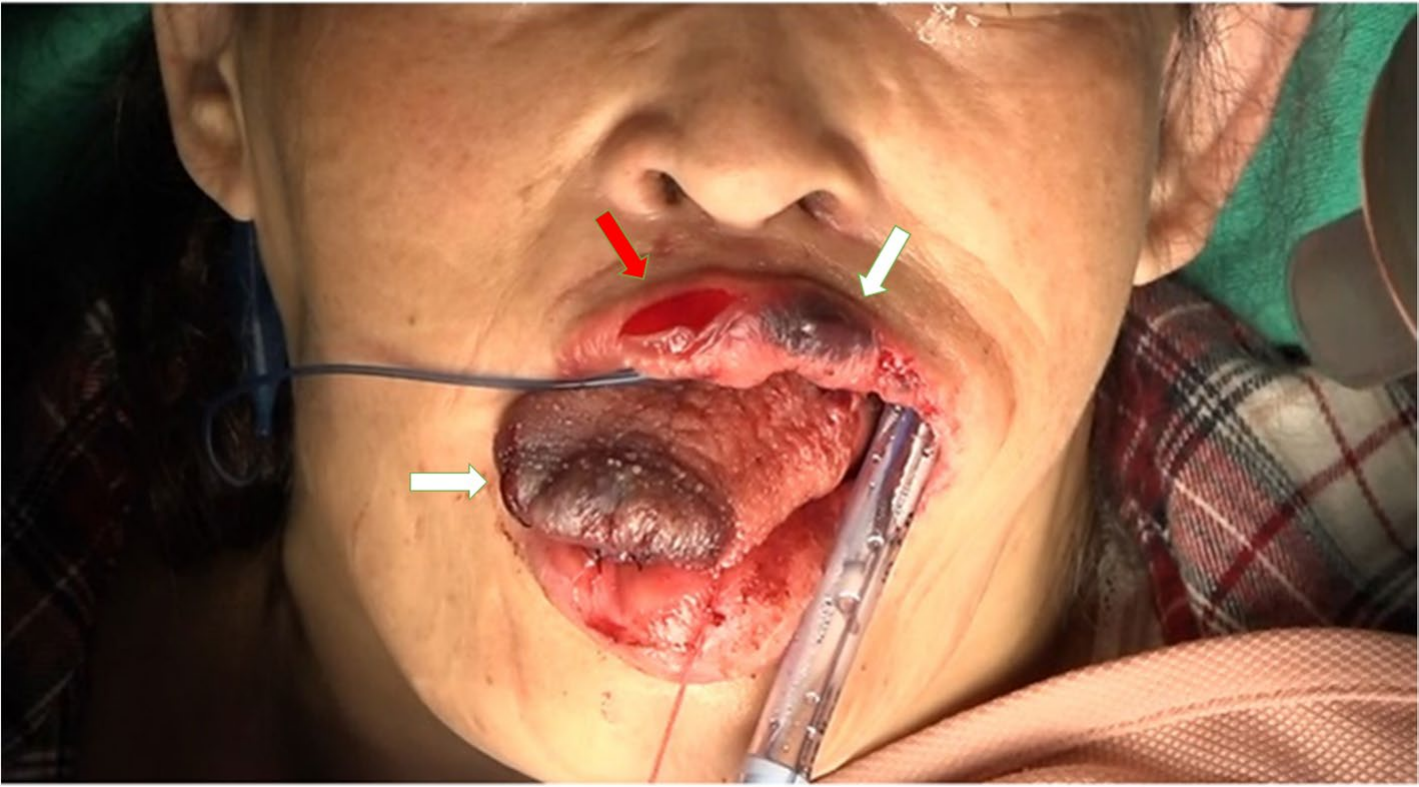
Vulnerable lips and tongue. Superficial laceration developed in the upper lip amid the transfer of the tracheal tube from the right to the left side of the mouth. Compression of surgical devices easily induced tissue swelling and hematoma. White arrows indicate hematomas in the upper lip and apex of the tongue. The red arrow indicates a superficial laceration of the upper lip

In the present case, the lips swelled and hematomas developed immediately after the start of surgery. The lips and tongue were likely to be fragile to physical stimuli. Amyloidosis may affect not only the tongue but also the lips [5]. Although a lip biopsy was not performed, it was likely that amyloidosis caused the lips to be vulnerable. In patients with oral amyloidosis, careful procedure is required to prevent lip injury during airway management.

## Data Availability

Not applicable.
